# B cell and/or autoantibody deficiency do not prevent neuropsychiatric disease in murine systemic lupus erythematosus

**DOI:** 10.1186/s12974-016-0537-3

**Published:** 2016-04-07

**Authors:** Jing Wen, Jessica Doerner, Samantha Chalmers, Ariel Stock, Haowei Wang, Maria Gullinello, Mark J. Shlomchik, Chaim Putterman

**Affiliations:** Department of Microbiology and Immunology, Albert Einstein College of Medicine, Bronx, NY USA; Department of Immunobiology, Yale University, New Haven, CT USA; Behavioral Core Facility, Department of Neuroscience, Albert Einstein College of Medicine, Bronx, NY USA; Department of Immunology, University of Pittsburgh, Pittsburgh, PA USA; Division of Rheumatology, Albert Einstein College of Medicine, F701N, 1300 Morris Park Ave, Bronx, NY 10461 USA

**Keywords:** Lupus, SLE, Neuropsychiatric SLE, B cells, Autoantibodies

## Abstract

**Background:**

Neuropsychiatric lupus (NPSLE) can be one of the earliest clinical manifestations in human lupus. However, its mechanisms are not fully understood. In lupus, a compromised blood-brain barrier may allow for the passage of circulating autoantibodies into the brain, where they can induce neuropsychiatric abnormalities including depression-like behavior and cognitive abnormalities. The purpose of this study was to determine the role of B cells and/or autoantibodies in the pathogenesis of murine NPSLE.

**Methods:**

We evaluated neuropsychiatric manifestations, brain pathology, and cytokine expression in constitutively (JhD/MRL/lpr) and conditionally (hCD20-DTA/MRL/lpr, inducible by tamoxifen) B cell-depleted mice as compared to MRL/lpr lupus mice.

**Results:**

We found that autoantibody levels were negligible (JhD/MRL/lpr) or significantly reduced (hCD20-DTA/MRL/lpr) in the serum and cerebrospinal fluid, respectively. Nevertheless, both JhD/MRL/lpr and hCD20-DTA/MRL/lpr mice showed profound depression-like behavior, which was no different from MRL/lpr mice. Cognitive deficits were also observed in both JhD/MRL/lpr and hCD20-DTA/MRL/lpr mice, similar to those exhibited by MRL/lpr mice. Furthermore, although some differences were dependent on the timing of depletion, central features of NPSLE in the MRL/lpr strain including increased blood-brain barrier permeability, brain cell apoptosis, and upregulated cytokine expression persisted in B cell-deficient and B cell-depleted mice.

**Conclusions:**

Our study surprisingly found that B cells and/or autoantibodies are not required for key features of neuropsychiatric disease in murine NPSLE.

## Background

Neuropsychiatric lupus (NPSLE) is a debilitating manifestation of systemic lupus erythematous (SLE), with a prevalence of approximately 40 % [1]. NPSLE manifestations include a broad spectrum of syndromes; headache, mood disorders, cognitive dysfunction, seizures, cerebrovascular diseases, and anxiety are the most common [2]. The pathogenesis of NPSLE is believed to be dependent upon whether the symptoms are focal or diffuse. Often, focal events such as cerebrovascular disease and seizures are associated with anti-phospholipid antibody-related hypercoagulability and thrombosis, while diffuse deficits, including depression and cognitive impairment, are mediated by as of yet unclear inflammatory pathways [3]. NPSLE can occur at any stage of disease [4]. The treatment options available are limited, with most patients treated empirically with immunosuppressive agents, corticosteroids, or symptomatic therapy [3].

The mechanisms underlying diffuse NPSLE development are not well understood; however, autoantibody-mediated brain damage and cytokine-induced CNS inflammation are believed to be important [5]. To date, several studies have examined whether specific subtypes of autoantibodies are involved in the pathogenesis (and can serve as biomarkers) of NPSLE. A role for anti-ribosomal P and anti-NMDA receptor autoantibodies in NPSLE has been proposed, but biomarker studies have not been entirely consistent [6]. Furthermore, while rituximab, an anti-CD20 monoclonal antibody used to deplete B cells in antibody-mediated diseases has shown some promise [7], a recent systematic review concluded that the evidence to date supporting its use in human NPSLE is relatively weak [8].

The MRL/lpr mouse strain is a validated murine lupus model, used extensively in the study of NPSLE [9]. Aside from the peripheral features such as autoantibody upregulation and severe kidney pathology, this strain also manifests cognitive deficits and depression-like behavior mimicking those found in human NPSLE [9]. Moreover, MRL/lpr mice exhibit an earlier onset of disease compared to NZB/W F1 and BXSB, and is one of the murine lupus strains with a greater prevalence of disease in females [9].

In order to investigate the potential role of B cells and autoantibodies in NPSLE, we utilized a B cell depletion strategy in MRL/lpr mice. A previous study examined the efficacy of rituximab in the MRL/lpr strain and found that B cell depletion was difficult to achieve, though after prolonged treatment, nephritis was ameliorated; however, NPSLE was not evaluated [10]. Since antibody-mediated cell depletion is markedly impaired in lupus [10], we employed a genetic approach to assess the roles of B cells in NPSLE manifestations, using constitutively deficient (JhD/MRL/lpr) and conditionally B cell-depleted (hCD20-DTA/MRL/lpr) lupus-prone mice.

## Methods

### Mice

JhD/MRL/lpr mice were generated as described previously [11] and were maintained as a homozygous colony. Human CD20-TamCre/MRL/lpr mice were created by backcrossing human CD20-TamCre/C57BL/6J mice, which possess an insertion of an IRES-Tam-Cre cassette into the 3′ UTR of the hCD20 locus [12], onto the MRL/lpr background (Jackson Laboratory, Bar Harbor, Maine) for more than seven generations. Human CD20-TamCre/MRL/lpr mice were maintained as heterozygotes via continuous breeding with MRL/lpr mice (Jackson Laboratory). Rosa26-Flox-Stop-DTA/MRL/lpr mice were generated by backcrossing Rosa26-Flox-Stop-DTA/C57BL/6J mice onto MRL/lpr mice for nine generations [13, 14]. The Rosa26-Flox-STOP-DTA locus contains a gene for the diphtheria toxin *α* chain (DTA), and the expression of the toxin is inhibited in the presence of the STOP cassette. Rosa26-Flox-Stop-DTA/MRL/lpr mice were maintained as homozygotes.

Human CD20-TamCre/MRL/lpr and Rosa26-Flox-Stop-DTA/MRL/lpr mice were crossed to generate the hCD20-TamCre-Rosa26-DTA/MRL/lpr (hCD20-DTA/MRL/lpr) mice, a tamoxifen inducible conditional B cell-depleted strain. To induce B cell depletion, hCD20-DTA/MRL/lpr mice were treated with intraperitoneal tamoxifen (0.2 mg/g weight) every other day starting at the age of 14 weeks, for five injections in total. After the last injection, mice were bled the next day for serum IgG and anti-double stranded DNA IgG ELISA. After confirming the depletion of antibodies, animals were allowed to rest for 5 days before neurobehavioral testing.

The MRL/MPJ strain (Jackson Laboratory) is a congenic background control strain for MRL/lpr mice. Unlike MRL/lpr mice, MRL/MPJ mice carry a normal (unmutated) *Fas* gene, and therefore do not exhibit a substantial autoimmune diathesis until a much older age. Since MRL/MPJ mice are genetically identical to MRL/lpr except in *Fas*, it is widely considered the most appropriate control for the latter lupus strain. At 4 months of age, while MRL/lpr mice display neuropsychiatric manifestations including depression-like behavior and cognitive deficits with the upregulation of inflammatory mediators, MRL/MPJ mice demonstrate normal emotionality and cognitive function with baseline brain cytokine expression [15–17].

For all strains employed in these studies, when not all mice of a particular strain were used for a given experiment, a subset of mice was chosen randomly. All animals were handled according to the approved animal protocol at the Albert Einstein College of Medicine.

### Genotyping

The detailed genotyping procedure for JhD/MRL/lpr mice by PCR was described previously [18]. For the human CD20-TamCre/MRL/lpr strain, positive litters were identified by the presence of Cre and ERT2 genes. The absence of the Rosa wild-type gene was used to screen for the Rosa26-Flox-Stop-DTA/MRL/lpr strain. The genotyping for these two strains was carried out by Transnetyx (Cordova, TN).

### Flow cytometry

Peripheral blood was obtained from the hCD20-DTA/MRL/lpr and JhD/MRL/lpr mice. MRL/lpr mice were used as positive controls. Blood samples were first lysed in red blood cell (RBC) lysis buffer and washed with ice-cold 2 % FBS. B cells were stained with anti-mouse CD19 conjugated to phycoerythrin (PE) for JhD/MRL/lpr, or allophycocyanin (APC) (BD Bioscience, San Jose, CA) for hCD20-DTA/MRL/lpr (the YFP gene in the latter strain is incompatible with red fluorochromes). The percentage of CD19 positive cells was gated out of single cell lymphocyte populations by flow cytometry (FACScalibur, BD Bioscience).

### ELISA

Anti-double stranded (ds) DNA antibodies in the serum and cerebrospinal fluid (CSF) were measured by ELISA, as previously described [19]. Total IgG in serum and CSF was measured by coating the plate with goat anti-mouse IgG (Southern Biotech, Birmingham, AL) overnight. Serum and CSF samples at a dilution of 1:500,000 and 1:500, respectively, were added to the plates for 2 h. Plates were then incubated with alkaline phosphatase-conjugated goat anti-mouse IgG (Southern Biotech) for 1 h, and developed with phosphatase substrate.

In lupus, the standard method most commonly used to measure anti-NMDAR antibody titers in both murine models [15, 20] and human studies [21–23] is by ELISA. Serum anti-NMDA receptor IgG ELISA was performed by coating the plate with multimeric peptide DWEYSVWLSN at 20 μg/ml, as described [15]. Serum samples were added to the plate at a dilution of 1:250 and detected with alkaline phosphatase-conjugated goat anti-mouse IgG (Southern Biotech). It is important, however, to recognize that using an ELISA-based method for measuring anti-NMDAR antibodies is not a standard neurological practice, and is not currently recommended for use in the clinical diagnosis of lupus.

### Neurobehavioral testing

Comprehensive behavior testing, including the Porsolt swim test, open field, object placement, and object recognition tests, were conducted following the procedures described previously [19], and further detailed below. JhD/MRL/lpr mice were tested at 7–12 weeks of age and CD20-DTA/MRL/lpr mice were tested at 16–18 weeks of age, with separate age matched controls for each group. Mice were randomly selected for testing when not all mice in a given genotype were subjected to a particular neurobehavioral assessment.

### Porsolt swim test

The Porsolt swim test is a standard and validated method used to evaluate depression-like behavior in rodents. Mice with depression-like behavior exhibit immobility, an indication of despair, while normal mice swim for the majority of the duration of the test. In the Porsolt swim test, each mouse was placed into a transparent cylindrical tank with water at 27 °C for 10 min. The first minute was not scored and in the following 9 min, immobility was scored manually with stopwatches. Each subject was digitally recorded using Viewer III software. Total immobility (as % total test duration) was calculated by the ratio of ([total time immobile in the water]/[total time scored]) × 100. The scorers were blind to the experimental groups.

### Open field test

The open field test was conducted in an arena (40 cm × 40 cm × 40 cm) with the center zone defined as 15 cm × 15 cm. General locomotor activity, including total track length, center track length, center time, and rears, were recorded by Viewer III software (Biobserve, Bonn, Germany) for 6 min.

### Novel object placement and novel object recognition tests

Object placement (OP) and object recognition (OR) tests were employed to assess spatial and visual recognition memory, respectively. In both tests, a training trial (trial 1) and a testing trial (trial 2) were conducted. In the OP trial 1, animals were given 5 min to explore two identical objects in different locations. After a 25-min retention interval, mice were returned to the same arena where one object was moved to a new location (trial 2). In the OR trial 1, animals were first allowed to explore two identical objects for 3 min. After a 90-min retention interval, animals were tested in the same arena where one object was replaced with a novel object (trial 2). The experimenters were blind to the experimental groups. The preference score (%) for object recognition and object placement tests was calculated as ([exploration time of the novel object (location)]/[exploration time of both objects (locations)]) × 100. The cutoff to define “pass” in the object placement and object recognition tests was 53 % [24, 25].

### Brain histology

Mice were sacrificed at 19 weeks of age. After extensive transcardial perfusing with cold PBS, the brains were dissected into right and left hemispheres. The right hemisphere of the brain was fixed in 4 % paraformaldehyde (PFA) for 24 h at 4 °C, and embedded in paraffin for sagittal sectioning. The left brain hemisphere including the region of the hippocampus was dissected, fixed in 4 % PFA for 24 h, followed by 30 % sucrose at 4 °C overnight, and utilized for frozen sections in a coronal plane. The remaining brain tissue was snap-frozen for RNA extraction.

### Immunofluorescence

For immunofluorescence staining, paraffin sections were incubated with mouse anti-glial fibrillary acidic protein (GFAP) (Millipore, Billerica, MA) or rabbit anti-mouse Iba1 (Chesterfield, VA) antibody, followed by donkey anti-mouse Alexa Fluor 594 or donkey anti-rabbit Alexa Fluor 488, respectively (Jackson ImmunoResearch, West Grove, PA). For albumin staining, paraffin sections were directly incubated with goat anti-mouse albumin-FITC (Alpha Diagnostic International, San Antonio, TX), and analyzed under a fluorescence microscope (Zeiss AxioObserver CLEM). Two sections were examined for each mouse brain. Quantitation of the intensity of Iba-1 and GFAP staining was performed by Image J.

### TUNEL staining

TUNEL staining was carried out by utilizing an in situ cell death detection kit-fluorescein (Roche, Indianapolis, IN) as described previously [26]. Slides were examined under a fluorescence microscope (Zeiss AxioObserver CLEM). One representative image was taken from each section, and the number of TUNEL positive cells in each section was counted. Two sections were analyzed for each mouse brain.

### Real-time PCR

RNA was isolated from the brain of MRL/lpr, JhD/MRL/lpr, hCD20-DTA/MRL/lpr, and MRL/MPJ mice at 19 weeks of age using RNEASY (Qiagen, Valencia, CA). Real-time PCR was performed in triplicate for selected genes including RANTES, MCP-5, IP-10, CXCL11, TNF, IFNβ, IRF7, and IFIT1 as described [19]. The relative expression of each gene was normalized to GAPDH, and the mean relative expression of each gene in the control group (MRL/MPJ) was set to 1.

### Statistical analysis

Statistical analysis was performed by Graphpad Prism 6 software. Data are displayed as mean ± standard error of the mean (SEM). The differences between two groups were calculated by an unpaired *t* test (two tailed). Non-parametric and non-normally distributed data were analyzed by the Mann-Whitney test. Fisher’s exact test was employed to compare the incidence of blood-brain barrier leakage between the two groups. Significance was considered as *p* < 0.05.

## Results

### B cells, antibodies, and autoantibody levels are negligible (JhD/MRL/lpr) or significantly reduced (hCD20-DTA MRL/lpr) in the serum and CSF

**Fig. 1:**
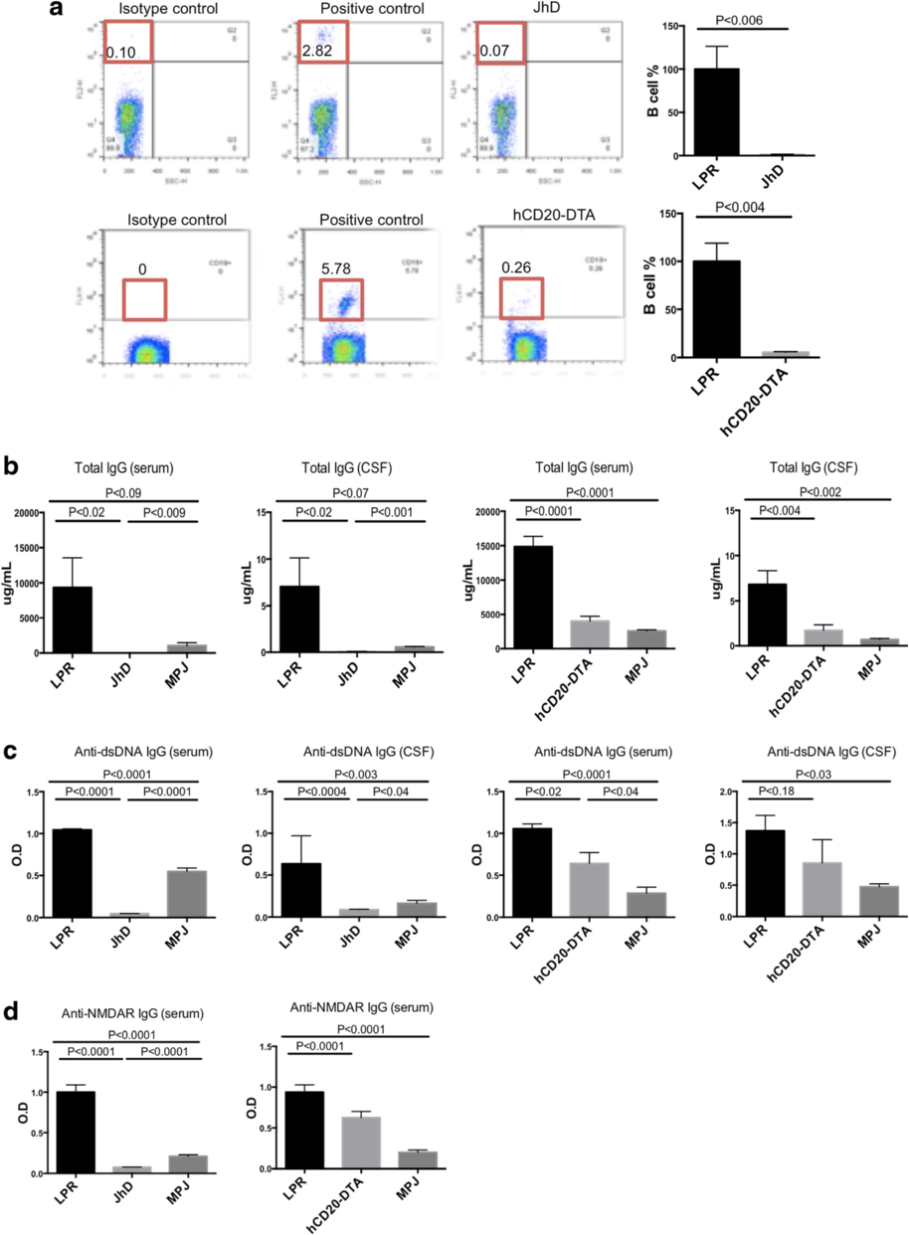
B cell counts and antibody titers are markedly reduced in the serum and CSF of JhD/MRL/lpr and hCD20-DTA/MRL/lpr mice. FACS analysis of CD19 positive cells in the peripheral blood of JhD/MRL/lpr and hCD20-DTA/MRL/lpr mice is shown in (**a**). The % CD19+ cells is provided in the *red box* in each panel. The MRL/lpr strain is used as a positive control. Total IgG levels (**b**) and anti-dsDNA IgG titers (**c**) in the serum and CSF of JhD/MRL/lpr and hCD20/MRL/lpr were measured by ELISA. Anti-NMDA receptor antibodies in the serum of JhD/MRL/lpr and hCD20-DTA/MRL/lpr mice are shown in (**d**). (JhD and control mice at 12–15 weeks of age: LPR mice, *n* = 5–8; JhD mice, *n* = 5–8; MPJ mice, *n* = 5–8; hCD20-DTA and control mice at 15 weeks of age: LPR mice, *n* = 7; hCD20-DTA mice, *n* = 7; MPJ mice, *n* = 7; except for the CSF ELISA experiments, where the number of mice in the LPR, hCD20-DTA ,and MPJ group were 7, 4, and 4, respectively). The CSF ELISA was done once; all other experiments were repeated twice, with similar results

To confirm B cell absence or depletion in JhD/MRL/lpr and hCD20-DTA/MRL/lpr mice, peripheral blood cells were stained for CD19 by flow cytometry. B cells were virtually absent in the JhD/MRL/lpr mice (Fig. 1a, upper right panel). Furthermore, a ~90 % reduction of B cells was observed in hCD20-DTA/MRL/lpr mice as compared to MRL/lpr mice (Fig. 1a, lower right panel). Confirming B cell deficiency in these strains, total IgG (Fig. 1b), anti-dsDNA IgG (Fig. 1c), and anti-NMDA receptor IgG (Fig. 1d) antibodies were virtually absent or significantly reduced in the serum and CSF of JhD/MRL/lpr and hCD20-DTA/MRL/lpr mice, respectively.

### JhD/MRL/lpr and hCD20-DTA/MRL/lpr mice demonstrate depression-like behavior and cognitive dysfunction

**Fig. 2:**
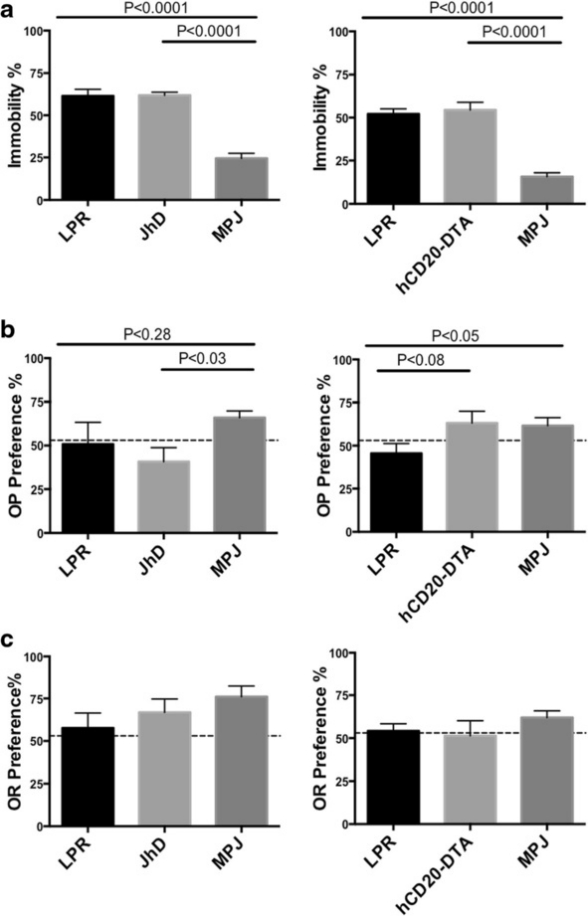
JhD/MRL/lpr and hCD20-DTA/MRL/lpr mice display depression-like behavior and cognitive dysfunction. The Porsolt swim test (**a**) was performed to evaluate behavioral despair in JhD/MRL/lpr and hCD20-DTA/MRL/lpr mice. (*Left panels*: LPR mice, *n* = 10; JhD mice, *n* = 17; MPJ mice *n* = 4; *Right panels*: LPR mice, *n* = 7; hCD20-DTA mice, *n* = 7; MPJ mice, *n* = 12). Object placement (**b**) and object recognition (**c**) tests were employed to assess cognitive function in JhD/MRL/lpr and hCD20-DTA/MRL/lpr mice. The *dotted line* indicates 53 % preference score. (*Left panels*: LPR mice, *n* = 5; JhD mice, *n* = 5; MPJ mice *n* = 5; *Right panels*: LPR mice, *n* = 7; hCD20-DTA mice, *n* = 7; MPJ mice, *n* = 10–11). Since testing affects subsequent performance in neurobehavioral analysis, all behavior tests in **a**–**c** were only conducted once. The JhD/MRL/lpr mice were tested at 7–12 weeks of age, and hCD20-DTA/MRL/lpr mice at 16–18 weeks of age. The control MRL/lpr and MRL/MPJ mice used for comparison were separately age matched to the JhD/MRL/lpr and hCD20-DTA/MRL/lpr strains, respectively

One of the distinctive clinical features of human NPSLE is mood disorder, especially depression [5]. Previously, we and others reported that MRL/lpr mice display profound depression-like behavior, exhibited by excessive immobility in the forced swim test as an indicator of behavioral despair [19, 27]. To investigate whether this depression-like behavior is B cell dependent, the Porsolt swim test was utilized in the B cell-deficient and B cell-depleted MRL/lpr mouse models. Surprisingly, both JhD/MRL/lpr and hCD20-DTA/MRL/lpr mice exhibited markedly increased immobility (50–60 % floating time) in water as compared to the control MRL/MPJ mice (15–25 % floating time), and identical to wild-type MRL/lpr mice (Fig. 2a). Thus, depression-like behavior persisted despite B cell deficiency or depletion in the MRL/lpr strain.

Cognitive impairment is another common manifestation of NPSLE in both humans and MRL/lpr mice [9]. Autoantibodies such as IgG anti-ribosomal P and anti-NMDA receptor have been suggested to play a role in cognitive deficits once the blood-brain barrier (BBB) is disrupted or circumvented [28, 29]. Thus, we investigated whether B cell-deficient or B cell-depleted lupus mice, which lack autoantibodies, would demonstrate cognitive dysfunction using object placement (spatial memory) and object recognition (visual memory) tests.

Mice preferentially explore novel objects and objects in novel locations. Thus, a failure to explore novel objects at greater than chance is an implicit measure of a failure to retain and/or recall the prior exposure to either an object or an object location, respectively, and is defined as cognitive (memory) impairment. JhD/MRL/lpr mice manifested spatial memory impairment (Fig. 2b), while their recognition memory was intact (Fig. 2c). Although hCD20-DTA/MRL/lpr mice trended toward improved spatial memory performance compared to MRL/lpr mice, the differences did not reach significance (Fig. 2b, right panel). hCD20-DTA/MRL/lpr mice did, however, exhibit cognitive deficits in the visual memory test (Fig. 2c). Our results suggest that, overall, evidence for cognitive dysfunction was present both for B cell-deficient or B cell-depleted MRL/lpr mice.

### JhD/MRL/lpr mice display increased general locomotion activity

**Fig. 3:**
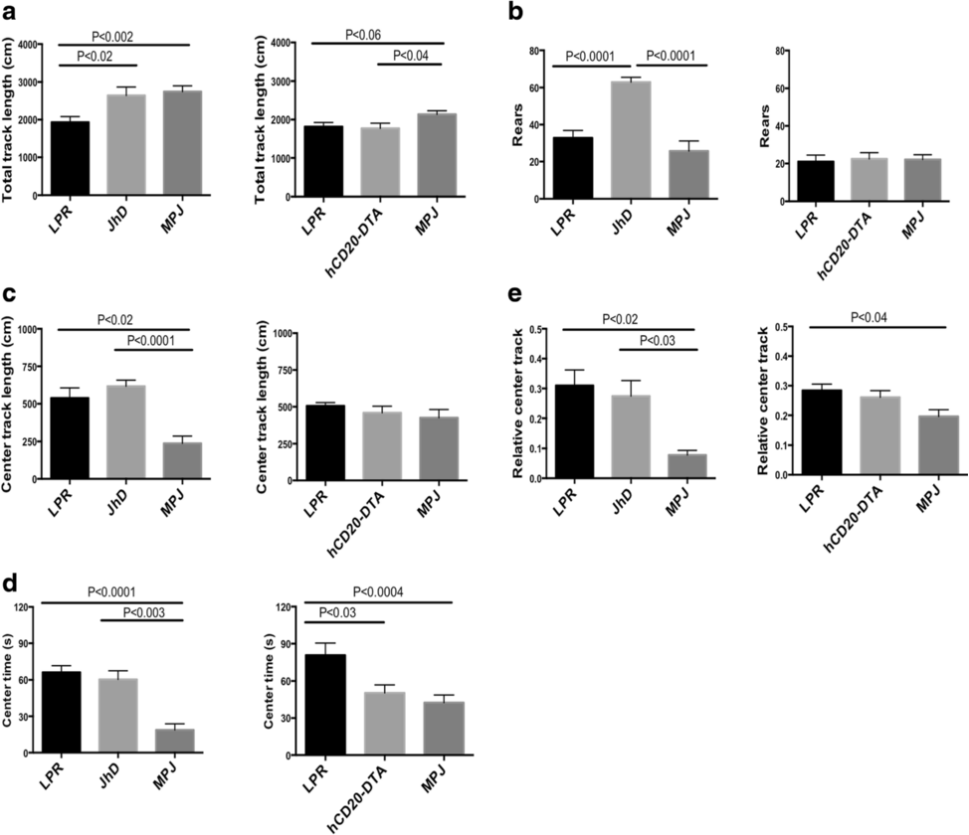
JhD/MRL/lpr mice exhibit increased general locomotor activity. General locomotor activity was assessed by open field test in JhD/MRL/lpr at 7–12 weeks of age and hCD20-DTA/MRL/lpr mice at 16 weeks of age including measurement of **a** total track length, **b** rears, **c** center track length (in cm), **d** center time (in seconds (s)), and **e** relative center track length (center track length/total track length). (*Left panels*: LPR mice, *n* = 11; JhD mice, *n* = 11–12; MPJ mice, *n* = 5–8; *Right panels*: LPR mice, *n* = 7; hCD20-DTA mice, *n* = 7; MPJ mice, *n* = 7–17). Open field tests were conducted once. The control MRL/lpr and MRL/MPJ mice used for comparison were separately age matched to the JhD/MRL/lpr and hCD20-DTA/MRL/lpr strains, respectively

Open field tests were carried out to assess locomotor and exploratory activity in B cell-deficient and B cell-depleted mice. Significantly increased total track length and number of rears were observed in JhD/MRL/lpr mice, indicating enhanced motor activity and exploration, respectively (Fig. 3a, b). hCD20-DTA/MRL/lpr mice, however, displayed behavior similar to MRL/lpr mice (Fig. 3a, b). The open field test can also assess risk-seeking behavior, as measured by the amount of time spent, or distance travelled, in the center of the testing arena. Interestingly, MRL/lpr and JhD/MRL/lpr mice demonstrated an increase in the center track length, relative center track length (center track length/total track length), and center time compared to MRL/MPJ mice, suggesting increased risk-seeking behavior (Fig. 3c–e). While the relative center track length was unaltered in hCD20-DTA/MRL/lpr mice compared to MRL/lpr mice (Fig. 3e), center time was significantly reduced (Fig. 3d). Thus, JhD/MRL/lpr mice exhibited enhanced overall motor activity and increased risk-seeking behavior. In contrast, total locomotion activity was unaltered and risk-seeking behavior was decreased in hCD20-DTA/MRL/lpr mice.

### JhD/MRL/lpr and hCD20-DTA/MRL/lpr mice exhibit increased BBB permeability

**Fig. 4:**
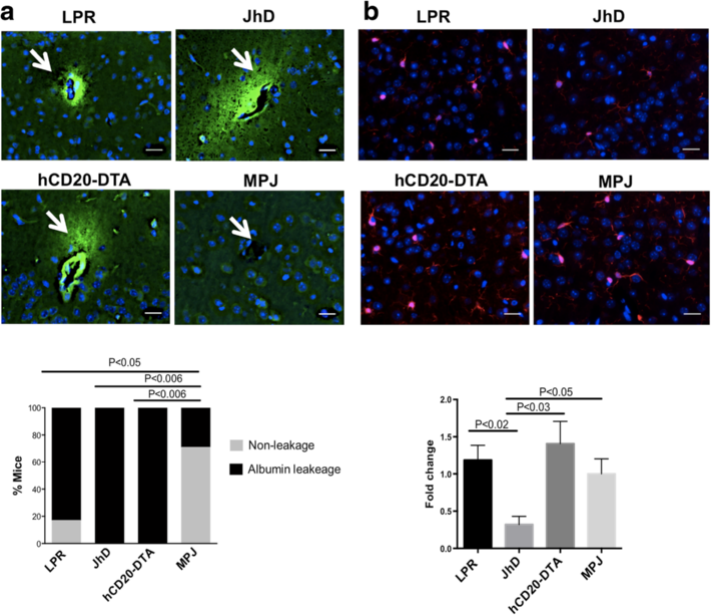
Blood-brain barrier permeability and microglial activation in B cell-deficient and B cell-depleted lupus mice. **a** Albumin staining was performed in the brain sections from MRL/lpr (*n* = 6), JhD/MRL/lpr (*n* = 4), hCD20-DTA/MRL/lpr (*n* = 7), and MRL/MPJ (*n* = 7) mice, all at 19 weeks of age. Representative images of albumin leakage around the vessels (indicated by *arrows*) in the cortex are shown. Percentages (%) of mice in each group that are positive for albumin leakage were displayed. Albumin staining was repeated twice, with similar results. **b** Iba-1 staining was performed on the brain sections of MRL/lpr (*n* = 6), JhD/MRL/lpr (*n* = 4), hCD20-DTA/MRL/lpr (*n* = 7), and MRL/MPJ (*n* = 7) mice, all at 19 weeks of age. Representative images of Iba-1 staining in the cortex are shown. Fold changes of fluorescence intensity are shown in the *bottom panel*, with the mean of MRL/MPJ set at 1. Iba-1 staining was repeated twice, with similar results. The scale bar in each image represents 20 μm

Disruption of the BBB has been observed in NPSLE patients, as demonstrated by gadolinium contrast-enhanced magnetic resonance imaging [30, 31]. Similarly, extravasation of IgG and other serum proteins into the CNS parenchyma was reported in MRL/lpr mice [32]. Here, we utilized albumin as an indicator of BBB leakage, and found extravasation of albumin predominantly in the cortex, periventricular areas, and cerebellum in the JhD/MRL/lpr, hCD20-DTA/MRL/lpr, and MRL/lpr strains. More than 80 % of mice in each of these strains demonstrated evidence of BBB disruption, as compared to the low baseline in MRL/MPJ mice (Fig. 4a). Similar results were obtained by employing immunofluorescent staining of the brain tissue for fibronectin, another marker for BBB leakage (data not shown).

### hCD20-DTA/MRL/lpr mice display comparable microglial activation to MRL/lpr

Microglia, as the major phagocytes of the CNS, have an important role in brain surveillance, as well as in homeostatic tissue remodeling and synaptic regulation [33]. With disruption of the BBB, peripheral inflammatory mediators may promote microglial activation. Immunofluorescent staining for Iba-1, a marker of microglial activation, was utilized for the purpose of exploring microglial activation following constitutive B cell deficiency or postnatal B cell depletion. Interestingly, no differences were observed in microglial activation among MRL/lpr, hCD20-DTA/MRL/lpr, and MRL/MPJ mice. However, JhD/MRL/lpr mice displayed reduced intensity of Iba-1 staining compared to the other three strains (Fig. 4b).

### JhD/MRL/lpr and hCD20-DTA/MRL/lpr mice reveal increased brain cell apoptosis

**Fig. 5:**
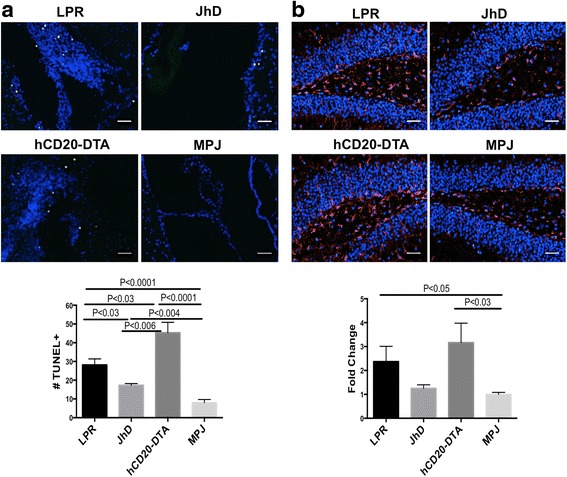
Brain cell apoptosis and hippocampal gliosis in JhD/MRL/lpr and hCD20-DTA/MRL/lpr mice. TUNEL and GFAP staining was performed in MRL/lpr (*n* = 6), JhD/MRL/lpr (*n* = 4), hCD20-DTA/MRL/lpr (*n* = 7), and MRL/MPJ (*n* = 7) mice, all at 19 weeks of age. Representative images of TUNEL staining in periventricular areas and choroid plexus are shown in (**a**, *top panels*). Quantitation of the average number of TUNEL positive cells in the two brain sections from each mouse is indicated in the *bottom panel*. **b** Representative images of GFAP staining in the hippocampus. Fold changes of fluorescence intensity are shown in the *bottom panel*, with the mean of MRL/MPJ set at 1. TUNEL and GFAP staining were repeated twice, with similar results. The scale bar in each image represents 40 μm

Cell apoptosis is one striking feature of the MRL/lpr mice brains, and is thought to be one of the underlying causes of the cognitive dysfunction [34, 35]. Thus, we investigated brain cell apoptosis in B cell-deficient and B cell-depleted lupus mice by TUNEL staining. The most common location of TUNEL positive cells in MRL/lpr mice was the periventricular area, as well as among the resident epithelial and the locally infiltrating inflammatory cells in the choroid plexus (Fig. 5a), but not in the hippocampus. Although JhD/MRL/lpr mice possessed significantly fewer TUNEL positive cells than MRL/lpr mice, these were elevated compared to MRL/MPJ (Fig. 5a). Surprisingly, hCD20-DTA/MRL/lpr mice exhibited significantly higher numbers of TUNEL positive cells, especially in cerebellum, compared to both MRL/lpr and MRL/MPJ mice, perhaps due to the pro-apoptotic properties of tamoxifen [36] (Fig. 5a). Thus, brain cell apoptosis was still present in B cell-deficient and B cell-depleted lupus mice.

### hCD20-DTA/MRL/lpr mice exhibit hippocampal astrogliosis

Astrogliosis, referring to astrocyte proliferation in response to CNS injury, is increased in the hippocampus of MRL/lpr mice [37]. To address whether astrocyte proliferation is affected by B cell deficiency, we examined expression of the astrocyte marker, GFAP, in the brain of JhD/MRL/lpr and hCD20-DTA/MRL/lpr mice. Upregulated hippocampal astrogliosis was observed in hCD20-DTA/MRL/lpr mice at a level similar to that found in MRL/lpr mice, while astrogliosis was less prominent in JhD/MRL/lpr mice (Fig. 5b).

### B cell deficiency or depletion in MRL/lpr mice modulates brain cytokine expression

**Fig. 6:**
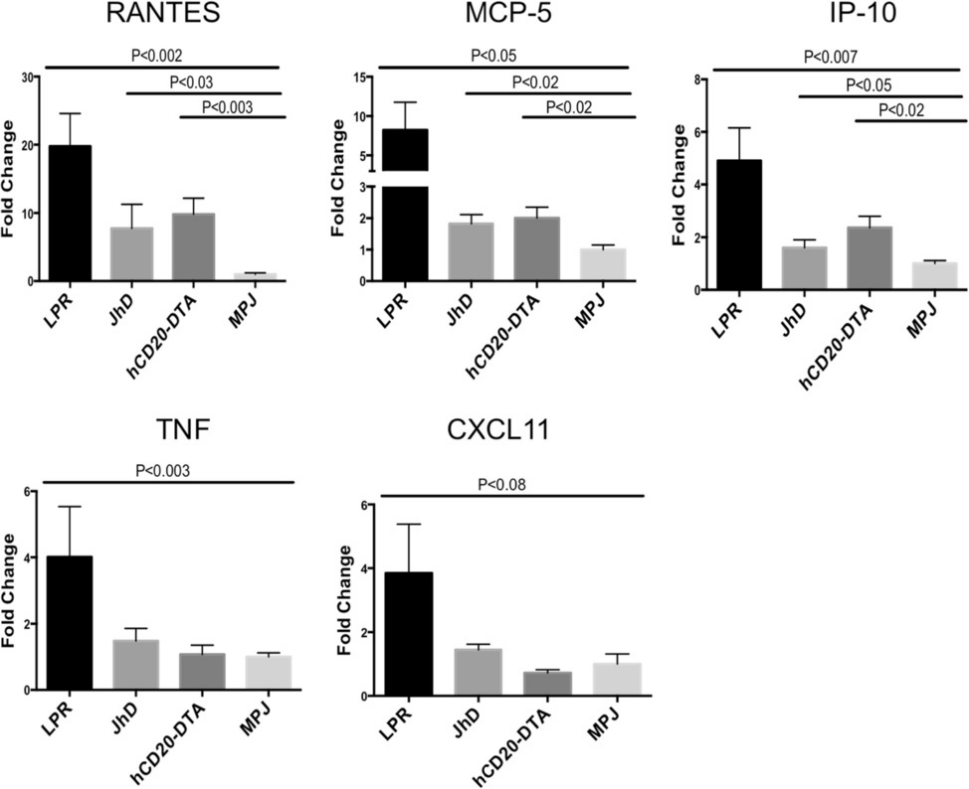
Cytokine expression in the brain of B cell-deficient and B cell-depleted lupus mice. Real-time PCR was performed on the brain RNA samples, extracted from randomly selected MRL/lpr (*n* = 6), JhD/MRL/lpr (*n* = 4), hCD20-DTA/MRL/lpr (*n* = 7), and MRL/MPJ (*n* = 7) mice, all at 19 weeks of age. Fold changes of each gene were calculated and normalized to the mean of each gene in MRL/MPJ group, which was set at 1. Real-time PCR was repeated twice, with similar results

Since multiple features of the NPSLE phenotype were preserved in JhD/MRL/lpr and hCD20-DTA/MRL/lpr mice, we investigated the effect of B cell deficiency or depletion on cytokine expression in the brains of lupus mice. Real-time qPCR for selected cytokines associated with SLE and/or NPSLE was carried out, including RANTES, IP-10, MCP-5, CXCL11, and TNF [19, 38–41]. An approximately two to fourfold suppression in the expression level of these cytokines was detected in brains of JhD/MRL/lpr and hCD20-DTA/MRL/lpr in comparison with MRL/lpr mice (Fig. 6). Nevertheless, RANTES, MCP-5, and IP-10 were still significantly upregulated; as compared to MRL/MPJ mice, RANTES expression was approximately tenfold, and MCP-5 and IP-10 expression approximately twofold higher. In contrast, the expression of CXCL11 and TNF was reduced to baseline levels in B cell-deficient or B cell-depleted mice (Fig. 6). We also examined the brain mRNA expression of classic interferon signature genes, including IFNβ, IRF7, and IFIT1. However, we found that there were no significant differences in the expression of any of these three genes among MRL/lpr, hCD20-DTA, JhD/MRL/lpr, and MRL/MPJ mice (data not shown). Thus, B cell deficiency or depletion reduced expression of some inflammatory mediators, but other cytokines were generally still expressed in the brains of JhD/MRL/lpr and hCD20-DTA/MRL/lpr mice at significantly higher levels than in the control MRL/MPJ strain.

## Discussion

In this study, we utilized JhD/MRL/lpr and hCD20-DTA/MRL/lpr mouse strains to investigate the role of B cells in the pathogenesis of NPSLE. Autoantibodies and B cells have either been directly linked or closely associated with lupus-associated neuropsychiatric manifestations [4, 42–45]. Evidence supporting the pathogenic role of autoantibodies can be found in studies which demonstrated that lupus-derived IgG autoantibodies directed against NMDAR [28], ribosomal P [46], and phospholipids [47] can induce neurobehavioral deficits, while in human lupus, the association of autoantibodies and neuropsychiatric symptoms has also been suggested by numerous studies [6]. Contrary to our expectations, however, we found that B cell deficiency or depletion does not ameliorate key manifestations of NPSLE, including depression-like behavior and cognitive dysfunction. Our findings therefore strongly suggest that B cells or circulating antibodies are not strictly required to fully reproduce the NPSLE phenotype.

Since B cell depletion required testing the mice when they were no longer young, any potential confounding effect of age on the behavioral phenotype needs to be considered. Nevertheless, while the wild-type and B cell-depleted MRL/lpr mice float excessively (>50 %) at 4 months of age, the age matched control MRL/MPJ mice displayed minimal floating (~16 %). Indeed, the lupus mice in our current study floated much more than older C57BL/6 mice tested previously (historical controls; data not shown). Thus, the reasonable conclusion would be that age did not significantly affect mobility in the swim test. Similarly in the other behavior tests as well, all animals are age matched, and therefore any possible effects of age were properly controlled for.

It is important nevertheless to note that B cell-deficient or B cell-depleted mice did not always display the same phenotype. In the neurobehavioral testing, JhD/MRL/lpr mice exhibited deficient spatial memory and increased total track length, while the hCD20-DTA/MRL/lpr displayed abnormal visual memory but preserved total track length. Furthermore, histopathological features characterizing the unmanipulated MRL/lpr strain including microglial activation, brain cell apoptosis, and astrogliosis were more prominent in the hCD20-DTA/MRL/lpr strain. This divergence can be explained by the different timing (i.e., congenital depletion) and relative completeness of B cell depletion (Fig. 1) occurring in the JhD/MRL/lpr strain. Furthermore, even though the brains from both strains were studied at the same age, the neurobehavioral assessment of hCD20-DTA/MRL/lpr was delayed by a few weeks due to tamoxifen administration. Indeed, some of the pathology observed in the hCD20-DTA/MRL/lpr strain could have been induced prior to administration of tamoxifen, or even after B cell depletion since autoantibodies did not totally disappear in this strain. Nevertheless, several key important features of the neuropsychiatric phenotype of murine (and human) lupus, including depression-like behavior (Fig. 2a) and permeabilization of the BBB (Fig. 4a), persisted despite B cell depletion and did not differ between the JhD/MRL/lpr and hCD20-DTA/MRL/lpr strains. We believe that the concurrence of these two features is not coincidental, as disruption of the neurovascular unit has been linked to non-lupus-related major depressive disorder as well [48]. Persistence of at least some of central features of murine NPSLE despite a marked reduction in, or ablation of, B cells and autoantibodies is an unexpected finding, which we believe is reported here for the first time. Nevertheless, certain histopathological features did variably improve, and cytokine expression was attenuated in B cell deficient mice, indicating that B cells and/or autoantibodies do affect the brain, albeit not in disrupting the BBB or contributing to depression-like behavior. Furthermore, we believe that another interesting and potentially revealing avenue for future studies will indeed be in attempting to understand the behavioral and pathophysiologic (e.g., microglial activation, brain cell apoptosis) differences between lupus mice with constitutive absence of B cells as compared to those with B cells depleted in later life.

The immune system plays a crucial role in cognition [49–51]. Microglia, as the major immune cells in the brain, function as phagocytes to clear cell debris and maintain surveillance and homeostasis in the central nervous system. Microglia, together with T cells, are implicated in hippocampal neurogenesis and spatial memory [52, 53]. Mice deprived of mature T cells displayed impaired hippocampal neurogenesis and cognitive dysfunction, which can be reversed by restoring T cells that recognized a specific brain antigen [53]. Furthermore, antibodies have been shown to affect brain growth and development [54]. Thus, although we are not aware of any direct evidence defining the role of endogenous B cells during development on cognition, these cells can possibly influence future cognitive function, either directly or through their interaction with other immune cells. We speculate that this might be one possible cause for the partial dependence of the neurobehavioral phenotype on the timing of B cell depletion.

The role autoantibodies play in the pathogenesis of NPSLE has yet to be fully clarified. Multiple brain reactive and non-brain specific antibodies are associated with NPSLE [6]. Anti-cardiolipin autoantibodies and lupus anticoagulant in particular have been strongly associated with cerebrovascular disease; anti-ribosomal P antibodies correlate with lupus psychosis, and anti-NMDA receptor antibodies have been associated with diffuse NPSLE manifestations [55]. This diversity in autoantibody specificities associated with NPSLE may represent the varied and complex nature of NPSLE manifestations, which plausibly can be related to multiple different autoantibodies. However, although some encouraging results were observed in clinical trials of NPSLE patients with the B cell-depleting therapy rituximab [56, 57], these studies only included a relatively small number of patients and were uncontrolled. Thus, the efficacy of rituximab as a therapeutic approach for NPSLE patients remains to be determined [57].

In murine models, several studies have demonstrated that autoantibodies can bind to brain specific antigens, inducing lupus-like pathologic behavior. Katzav et al. found that anti-ribosomal P antibodies deposited in the limbic system (piriform cortex, hippocampus, and cingulate cortex) can produce depression-like behavior [46]. Similarly, a recent study showed that anti-ribosomal P antibodies purified from NPSLE patients bound to hippocampal neurons in the brains of healthy mice, leading to memory impairment [29]. Moreover, Kowal et al. demonstrated that following compromise of the BBB, anti-NMDA receptor antibodies can induce neuronal cell death resulting in cognitive dysfunction [28]. Specifically, these antibodies induced apoptosis in the CA1 region of the hippocampus [28]. Nevertheless, to our surprise, we found that B cell-deficient and B cell-depleted MRL/lpr mice continued to exhibit features of the characteristic NPSLE phenotype of this strain. Both JhD/MRL/lpr and hCD20-DTA/MRL/lpr mice demonstrated depression-like behavior and cognitive deficits similar to unmanipulated MRL/lpr mice, as well as BBB disruption, brain cell apoptosis, and cytokine upregulation. This observation can be explained in several ways. First, although the abrogation of BBB may indeed be a prerequisite for autoantibodies to access the brain, the opening of BBB is usually transient, and eventually, the autoantibodies that bypass the BBB play a less important role in disease initiation than other inflammatory mediators within the CNS. Second, the strains of mice were different; autoimmune MRL/lpr mice (a strain with spontaneous disease and thus more similar to human SLE) are characterized by a complex dysregulation of cytokines and antibodies, while previous studies addressing the role of pathogenic antibodies in murine NPSLE used non-autoimmune strains, and tested whether manipulations elicited acute behavioral or histologic alterations.

We found that disruption of the BBB persisted despite acute B cell deficiency or depletion. This breached BBB can trigger an inflammatory cascade, modulating astrocyte-neuron crosstalk and altering the release of neurotransmitters, which may have contributed to the observed behavioral abnormalities. Increased brain cell apoptosis among JhD/MRL/lpr, hCD20-DTA/MRL/lpr, and MRL/lpr mice may be another consequence of BBB disruption. While the more marked apoptosis observed in the hCD20-DTA as compared to the JhD/MRL/lpr strain may have been contributed by tamoxifen [36], the fact that both MRL/lpr and to a lesser degree JhD/MRL/lpr had significantly increased brain cell apoptosis as compared to the MRL/MPJ mice suggests that this process is a characteristic of (if not a contributor to) NPSLE in this strain, which is not eliminated by a reduction in the numbers of B cells.

The role of cytokines in NPSLE is supported by numerous studies. Although SLE is characterized by high titers of autoantibodies, the onset of NPSLE can occur at any stage of the disease, even prior to a significant rise in autoantibody titers [15]. Furthermore, the severity of NPSLE may not correlate with autoantibody titers [58]. MRL/lpr mice develop depression-like behavior before the peak of autoantibody production or evidence of systemic disease [19]. Cytokine dysregulation, however, occurs as early as 1–4 weeks of age [59–62]. We found decreased expression of RANTES, MCP-5, IP-10, CXCL11, and TNF following B cell deficiency or depletion in MRL/lpr mice. Nevertheless, at least RANTES, MCP-5, and IP-10 remained significantly elevated in B cell-deficient and B cell-depleted mice in comparison with MRL/MPJ mice. This observation suggests that these cytokines (or others not tested for), probably derived from CNS resident cells such as astrocytes and microglia, may actually be the key effectors in initiating the pathogenesis of NPSLE—at least in the absence of B cells and/or autoantibodies. This observation is consistent with several clinical studies that found that increased levels of RANTES, IP-10, and MCP-5 in the CSF correlate with NPSLE manifestations [38–41]. Furthermore, the persistence of the NPSLE phenotype in JhD/MRL/lpr and hCD20-DTA/MRL/lpr mice may indicate that mood and cognition are very sensitive to cytokine abnormalities, and therefore, a robust NPSLE phenotype is manifested even with lower brain cytokine levels than in the MRL/lpr strain.

Besides the role cytokines play in CNS abnormalities, other potential contributors may also need to be taken into consideration. Long-lived plasma cells secreting autoantibodies are resistant to B cell depletion [10]; however, these would be absent in the B cell-deficient strain (JhD/MRL/lpr), and thus, these plasma cells are unlikely to be the early mediators of NPSLE. Additionally, Chan et al. reported that although tenfold reductions in CD4^+^ and CD8^+^ T cells were observed in B cell-deficient mice (JhD/MRL/lpr), a few T cells were persistent [18], suggesting a potential role for T cells as well. Furthermore, gut microbiota has recently been suggested to be involved in brain function including anxiety, mood, cognition, and pain [63]. Therefore, whether B cell deficiency or depletion modulates gut microbiota will be an interesting question to explore. Finally, Fas and Fas ligand are expressed in the CNS, and one may ask whether the neuropsychiatric symptoms seen in MRL/lpr mice are mediated by abnormal Fas signaling [64], for example, by altering apoptosis in microglia and/or other brain cell types. Nevertheless, since NPSLE manifestations also appear in other, non-Fas-mediated murine lupus models [9], autoimmunity is the most likely mechanistic explanation in the MRL/lpr model as well.

Thus, there are several potential mechanisms to explain the persistence of neuropsychiatric lupus in MRL/lpr mice despite a significant decrease in, or depletion of, B cells and autoantibodies, including pathways involving cytokines, microglial activation, neurotransmitters, etc. We acknowledge that while the differential effects on cytokines reported above are interesting, these studies are preliminary. Moreover, the additional possible mechanisms will need to be further explored. Indeed, considering the complex immune abnormalities present in SLE, we believe that the neurobehavioral deficits seen in murine lupus strains (and in human disease) are most likely due to the combined contributions of more than a single mechanism. The lack of strict dependence of neuropsychiatric lupus on the presence of B cells and autoantibodies is nonetheless a novel and surprising observation, and additional investigation outside the scope of the present study will be required in the future to continue to define the relevant molecular pathways.

## Conclusions

Our data collectively indicate that lack of B cells and autoantibodies in MRL/lpr mice does not prevent the development of key features of NPSLE; this is most convincingly shown by the neurobehavioral deficits observed in JhD/MRL/lpr mice that lacked B cells from birth. Furthermore, among the cytokines studied, we found that RANTES, IP-10, and MCP-5 were persistently elevated in B cell-deficient and B cell-depleted MRL/lpr mice, suggesting a possible role as effectors of NPSLE pathogenesis. Future studies can include a more comprehensive characterization of the cytokines produced in B cell-deficient and B cell-depleted mice and identification of their cellular origins within the CNS, and further exploration of the contribution of T cells and microbiota. It will be interesting to test whether intracerebral cytokine administration alone can induce a NPSLE-like phenotype. If in fact that is the case, such a mediator may provide a valuable therapeutic target for this challenging lupus end-organ manifestation.
